# The effect of targeted wide age range SIAs in reducing measles incidence in the African Region

**DOI:** 10.11604/pamj.supp.2017.27.3.12176

**Published:** 2017-06-21

**Authors:** Balcha Masresha, Richard Luce, Regis Katsande, Amadou Fall, Meseret Eshetu, Richard Mihigo

**Affiliations:** 1 World Health Organization, Regional office for Africa, Immunisation and Vaccine Development Program, Brazzaville, Republic of Congo; 2 World Health Organization, Intercountry Support Team for Central Africa, Libreville, Gabon; 3 World Health Organization, Intercountry Support Team for East and Southern Africa, Harare, Zimbabwe; 4 World Health Organization, Intercountry Support Team for Western Africa, Ouagadougou, Burkina Faso

**Keywords:** Measles, elimination, supplemental, immunisation

## Abstract

Periodic measles supplemental immunisation activities (SIAs) increase population immunity and thereby reduce the pool of accumulated susceptible children. They are typically conducted every 2 – 4 years, and most often target children up to five years of age. Between 2012 and 2015, after surveillance data indicated a shift in the epidemiological profile of measles towards older age groups, 11 countries were supported to conduct wide age range SIAs based on their local epidemiological patterns. Six other countries conducted SIAs with measles-rubella vaccines targeting ages 9 months to 14 years as an initial step of introducing rubella vaccine into the immunization program. In subsequent years, the incidence of confirmed measles dropped significantly in 13 of the 17 countries reviewed. The findings emphasize the importance of well-functioning surveillance systems, and the benefits of using of surveillance data to determine the specific target age-range for periodic SIAs to accelerate progress towards measles elimination.

## Introduction

Member States in the African Region of the WHO have been implementing strategies for measles control since 2001. These efforts include improving routine measles immunisation coverage, providing supplemental doses of measles vaccines through periodic mass vaccination campaigns, and conducting intensified case-based disease surveillance supported with serological laboratory confirmation and genotyping of circulating measles strains [1].

In 2011, the sixty-first session of the WHO Regional Committee for Africa adopted a measles elimination goal to be achieved by 2020. The targets for the elimination goal are: confirmed measles incidence of less than 1 case per million population at national level; at least 95% routine measles immunization coverage at national level and in all districts; at least 95% coverage in all measles SIAs, and attaining the targets for the two main surveillance performance indicators. These are (1) Non-measles febrile rash illness rate: (target: at least 2 per 100,000 population) and (2) Proportion of districts reporting at least 1 suspected case of measles with a blood specimen per year: (Target: at least 80%) [2].

Initial measles catch-up campaigns were conducted between 2001 and 2006 in 43 countries in the Region targeting children from 9 months up to 14 years of age with the objective of ensuring that the entire childhood and young adolescent population is vaccinated against measles. Periodic follow-up campaigns are organized every 2 – 4 years to reach children starting from 6 or 9 months of age and up to 47 or 59 months of age, in order to provide a supplemental dose of measles vaccine to protect unvaccinated children born since the most recent mass vaccination campaign. The WHO guidelines for planning and implementing SIAs indicate that the upper age limits for measles and rubella SIAs should be determined for based on disease epidemiology and the estimated susceptibility by age [3].

We conducted this analysis in order to examine the effect of wide-age range SIAs on measles incidence.

## Methods

Mass vaccination campaigns or supplemental immunisation activities (SIAs) are typically conducted as nationwide activities over a period of 5 – 10 days often. SIA administrative coverage at the subnational and national level is calculated by tallying the numbers of administered doses and dividing by the target population [3].

According to the Regional surveillance standards (World Health Organization. Regional Office for Africa. African Regional guidelines for measles and rubella surveillance- Revised April 2015), the measles case definition used to report suspected cases in the case based surveillance system is: fever and generalized maculopapular rash plus one of the following clinical symptoms: cough, runny nose, or red eyes. For each suspected measles case, an investigation form is completed, a blood specimen is collected and sent to the national laboratory for measles-specific immunoglobulin M (IgM) antibody testing.

Suspected measles cases are confirmed by laboratory when there is serological confirmation of recent measles virus infection (measles IgM positive) and the case has not received measles vaccination in the 30 days preceding the specimen collection. Cases may also be confirmed by epidemiological linkage or on clinical grounds. Epidemiological linkage is used once an outbreak is confirmed in a district and is defined as a suspected measles case that has not had a specimen taken for serologic confirmation and is linked (in place, person and time) to lab confirmed cases; A clinically compatible case of measles is a suspected measles case that fits the epidemiological case definition, but has not had a blood specimen taken for serologic confirmation and is not linked epidemiologically to any measles outbreak.

Surveillance performance is monitored using standard performance indicators. The two principal indicators are: non-measles febrile rash illness rate (target of at least 2 per 100 000 population) and the proportion of districts that have investigated at least one suspected case of measles with blood specimen per year (target of 80% or more per year). The incidence of confirmed measles is calculated as a rate per million, by dividing the total number of confirmed measles cases (by laboratory, epidemiological linkage and clinically) by the total population.

We identified 17 countries in the African Region where wide-age range measles or measles-rubella (MR) mass vaccination campaigns were conducted from 2012 - 2015. Of these, 6 countries (Burkina Faso, Ghana, Senegal, Rwanda, Tanzania and Zimbabwe) conducted measles-rubella catch-up SIAs targeting children 9 months to 14 years during this period. The other eleven countries (Angola, Benin, Chad, Cote d’Ivoire, Democratic Republic of Congo, Mali, Mauritania, Namibia, Niger, Togo and Zambia) organized wide age range measles SIAs based on the epidemiological pattern of measles in the countries. Namibia and Zambia carried out national MR catch-up SIAs in 2016 targeting children 9 months to 14 years, in addition to the wide-age range measles SIAs conducted in 2012 in response to measles outbreaks involving older children. Zimbabwe had done an outbreak response campaign in 2010 targeting children 6 months to 9 years.

We analysed surveillance performance, and incidence levels across these countries in the years before and after the wide-age range SIAs. Democratic Republic of Congo was excluded from this analysis since only a small fraction of reported measles cases was investigated through the case based surveillance system.

## Results

The wide-age range SIAs conducted between the years 2012 - 2015 are shown in Table 1. The administrative coverage and coverage from post-campaign coverage surveys are shown for comparison. In 6 countries, post-campaign coverage surveys were not done.

**Table 1:** Wide age range measles and measles-rubella SIAs in AFR, 2012 - 2016

Country	Year	Type of SIAs	Age group targeted	Number of children vaccinated	Administrative coverage (% of target)	% districts with 95% or above coverage	Coverage survey result (% of target)
Angola	2014	Measles follow-up	6 mo – 9 yrs	9,169,335	117%	84%	97.2%
Benin	2014	Measles follow-up	9 mo - 9 yrs	3,030,145	100.7%	81.8%	96.5%
Chad	2014	Measles follow-up	6 mo - 9 yrs	2,549,188	103.4%	94.1%	
Cote d'Ivoire	2014	Measles follow-up	6 mo - 9 yrs	9,640,512	92.1%	94.5%	94.7%
Ghana	2013	MR catch-up SIAs	9 mo - 14 yrs	11,062,605	99.0%	70.0%	95.7%
Burkina	2014	MR catch-up SIAs	9 mo - 14 yrs	8,517,508	106.8%	100.0%	
Mali	2015	Measles follow-up	9 mo - 14 yrs	9,312,619	112.3%	90.5%	93.5%
Mauritania	2014	Measles follow-up	9 mo - 14 yrs	1,489,563	105.1%	92.3%	
Namibia	2012	Measles follow-up	6 mo - 14 yrs	885,259	91.1%	100.0%	97%
	2016	MR catch-up SIAs	9 mo - 39 yrs	1,908,193	103%	76.9%	
Niger	2012	Measles follow-up	9 mo - 14 yrs	7,736,066	101.6%	95.2%	96.50%
Rwanda	2013	MR catch-up SIAs	9 mo - 14 yrs	4,391,081	102.6%	90.0%	98.0%
Senegal	2013	MR catch-up SIAs	9 mo - 14 yrs	6,097,155	101.4%	76.0%	97.0%
Tanzania	2014	MR catch-up SIAs	9 mo - 14 yrs	20,529,629	97.0%	59.3%	89.0%
Togo	2013	Measles follow-up	9 mo - 10 yrs.	1,641,635	96.3%	83.0%	
	2015	Measles follow-up	9 mo - 10 yrs.	820,335	98.6%	94%	
Zambia	2012	Measles follow-up	6 mo - 14 yrs	7,503,515	116.5%	93%	96%
	2016	MR catch-up SIAs	9 mo - 14 yrs	7,741,505	108.1%	96.5%	
Zimbabwe	2015	MR catch-up SIAs	9 mo - 14 yrs	5,337,029	102.6%	100%	94%

All 16 countries reported administrative coverage at the national level of greater than 95% except for Cote d’Ivoire and Namibia in 2012 where coverage was 92% and 91% respectively. At the sub-national level, the percentage of districts achieving 95% administrative coverage ranged from 59% in Tanzania to 100% in Zimbabwe. All post-campaign coverage surveys except Mali and Tanzania estimated coverage of 95% or greater. The country level incidence rate of confirmed measles is plotted for the years 2010 to 2016 (Table 2). The years in which the wide-age range campaigns were conducted are shaded in the table. The years 2010, 2011 and 2016 were included in the table for comparison purposes. Eight of the 16 countries (Angola, Burkina Faso, Chad, Mali, Mauritania, Namibia, Tanzania and Zambia) have not met the targets for the two main surveillance performance indicators for at least 3 of the 5 years analysed. Eight countries (Benin, Cote d’Ivoire, Ghana, Niger, Rwanda, Senegal, Togo, Zimbabwe) achieved both targets for at least 3 of the 5 years (Table 3).

**Table 2:** Incidence of confirmed measles by country by year, 2012 - 2016

	2010	2011	2012	2013	2014	2015	2016
Angola	60.2	8.0	204.7	285.3	**522.2**	2.7	1.3
Benin	14.4	45.8	29.6	48.1	**77.0**	3.9	8.2
Burkina Faso	27.4	16.7	42.6	24.9	**24.2**	5.4	11.7
Chad	19.8	8.7	13.8	12.9	**95.8**	30.8	10.7
Cote d'Ivoire	22.5	26.3	6.0	2.0	**2.0**	1.6	2.1
Ghana	1.5	5.3	13.9	**12.0**	5.3	1.8	1.8
Mali	67.2	1.4	22.2	17.8	16.4	**13.5**	5.8
Mauritania	17.0	57.0	1.2	0.9	**4.0**	0.3	3.4
Namibia	656.4	35.9	**43.6**	21.8	292.2	96.4	**4.9**
Niger	22.1	48.9	**19.1**	46.6	17.5	35.9	30.8
Rwanda	6.2	2.8	7.7	**1.7**	0.5	0.1	5.0
Senegal	36.7	1.7	4.1	**1.0**	2.7	4.1	10.9
Tanzania	2.0	35.3	16.4	0.4	**1.3**	0.4	0.7
Togo	24.1	26.0	39.5	48.1	**24.7**	2.6	4.1
Zambia	107.3	97.9	**2.9**	0.1	1.1	1.4	**0.4**
Zimbabwe	**725.0**	0.2	0	0.2	5.1	**0.1**	0.1

**Table 3:** Country measles surveillance performance as per the two principal monitoring indicators, 2012 - 2016

	Non-measles Febrile Rash Illness Rate per 100,000 population by year (target > 2:100,000)					Proportion of districts reporting at least 1 case with blood specimen by year (target > 80%)				
	**2012**	**2013**	**2014**	**2015**	**2016**	**2012**	**2013**	**2014**	**2015**	**2016**
Angola	2.4	1.1	1.4	1.9	0.8	69%	70%	77%	61%	41%
Benin	2.6	2.3	1.4	1.9	2	86%	100%	90%	75%	79%
Burkina Faso	3.3	1.9	2.3	0.6	0.8	44%	64%	86%	100%	100%
Chad	0.3	0.6	0.4	1.5	1.9	100%	90%	95%	77%	79%
Cote d’Ivoire	3.8	2.2	2	2.5	4.7	87%	87%	87%	79%	80%
Ghana	4.8	2.8	3.3	3.5	2.6	100%	100%	100%	100%	100%
Namibia	16	6.8	16.5	45.6	10.9	71%	82%	91%	100%	100%
Mali	1.4	1.4	1.8	2.3	1.8	47%	26%	55%	28%	34%
Mauritania	2	0.6	3	1	0.9	73%	59%	73%	75%	85%
Niger	2	2.4	0.8	2.4	1.3	83%	100%	98%	100%	98%
Rwanda	6.5	6.4	3.7	3.6	7.3	100%	100%	94%	94%	94%
Senegal	2.2	3.8	6.8	5.2	5.1	100%	100%	89%	100%	91%
Tanzania	1.2	0.1	1.7	0.8	1.9	94%	81%	100%	99%	101%
Togo	2.9	3.5	3.3	4	7.5	97%	92%	92%	95%	98%
Zambia	2.4	0.6	1.9	2.3	2.3	58%	32%	53%	75%	79%
Zimbabwe	2.1	4.5	15.5	2.5	3.1	92%	98%	100%	86%	94%

All countries reported a decrease in confirmed measles incidence in the calendar year immediately after the mass campaigns except for Senegal and Niger. Measles incidence declined by 99.5% in Angola after the 2014 SIA, representing the largest decrease.

## Discussion

In the years after the wide age range mass campaigns, Angola, Benin, Burkina Faso, Chad, and Togo (with pre-SIA incidence of greater than 20 per million), as well as Ghana, Mali, Mauritania, Rwanda and Tanzania (pre-campaign incidence of less than 15 per million) all experienced a pronounced decline in confirmed measles incidence that was sustained for at least 2 years. On the other hand, Cote d’Ivoire, Namibia, Niger and Senegal did not have such a decrease in measles incidence as compared to the preceding years. Cote d’Ivoire and Senegal had incidence levels of less than 5 per million for a number of years. Niger had incidence levels of more than 20 per million and Namibia reported more than 200 cases per million in 2014.

The lack of consistently effective measles surveillance in countries such as Angola, Burkina Faso and Chad, may suggest that the incidence is underestimated and confound interpretation of surveillance data. Nevertheless surveillance performance did not change significantly from the pre to post-campaign period, and so comparisons are still useful.

Although administrative coverage was greater than 90% for all SIAs, the subnational data suggest that high coverage was not homogeneous and that areas of non-vaccinated children persist. For example, at the national level, Senegal reported coverage of over 100% and the post-campaign coverage survey estimated 97% coverage; however only 76% of districts had administrative coverage of 95% or more. This may explain why incidence did not decline in the year after the SIAs since measles transmission could continue among susceptible individuals.

It has been shown that the combined efforts of increased routine immunization coverage, and periodic mass campaigns have led to a significant decline in the measles disease burden and estimated measles deaths in the Region [4]. Countries have documented a decline in the frequency and size of measles outbreaks, and also a change in the epidemiological pattern of measles cases, with an increasing proportion of school-aged children and young adults. This was noted particularly in countries with MCV-1 coverage of more than 50% [5]. Such a pattern was also noted in other countries and WHO Regions [6, 7].

Further analysis of data from surveillance will be needed to look at the changes in the age pattern of confirmed measles cases in these countries, and comparing incidence changes in follow-up campaigns targeting only under-fives against the wide age range campaigns. This analysis has not attempted to look at the impact of increasing coverage in the routine immunization program on measles circulation, and the homogeneity of coverage of SIAs towards the decline in incidence of disease.

## Conclusion

Combined with routine immunization of target children, SIAs are an essential tool in building up of population immunity. The results of the analysis of wide age range measles SIAs on measles incidence in selected countries demonstrates the important role they play in achieving progress toward the regional measles elimination objectives. High quality measles surveillance is important to monitor epidemiological trends to aid in determining appropriate SIA frequency and targeting of affected age groups, which should be determined on a case-by-case basis prior to each SIAs for maximum impact. The introduction of measles-rubella (MR) vaccine provides an important opportunity to conduct initial wide-age range catch-up SIAs reaching children up to 14 years of age. There is a clear need to improve SIAs coverage to ensure more homogenous high coverage in all districts. In addition, it will be important for countries to introduce a second dose of measles vaccine in their routine programs to further improve population immunity.

### What is known about this topic

- Periodic supplemental immunisation activities (SIAs) are essential in order to close immunity gaps created through suboptimal routine immunisation coverage, and have contributed to the significant reduction of disease burden and estimated measles deaths;
- The epidemiological pattern of measles has been shifting since the start of implementation of measles mortality reduction strategies in the African region, with an increasing proportion of school-age children being affected in many countries;
- WHO guidelines recommend that the target age group for periodic SIAs should be guided by the specific epidemiological patterns in target countries.

### What this study adds

- The determination of target age groups for periodic measles SIAs can be guided by using available case based surveillance data;
- 12 of the 14 countries that conducted wide age range campaigns reported a significant decline in confirmed measles incidence in the calendar year immediately after the SIAs;
- The homogeneity of SIAs coverage determines whether or not sustained decline in measles incidence is achieved in the years subsequent to the SIAs.

## Competing interests

The authors declare no competing interest.
